# Antiviral effects of nisin, lysozyme, lactoferrin and their mixtures against bovine viral diarrhoea virus

**DOI:** 10.1186/s12917-019-2067-6

**Published:** 2019-09-05

**Authors:** Joanna Małaczewska, Edyta Kaczorek-Łukowska, Roman Wójcik, Andrzej Krzysztof Siwicki

**Affiliations:** Department of Microbiology and Clinical Immunology, Faculty of Veterinary Medicine, University of Warmia and Mazury in Olsztyn, Oczapowskiego Street 13, 10-718 Olsztyn, Poland

**Keywords:** Nisin, Lysozyme, Lactoferrin, Bovine viral diarrhoea virus, Antiviral

## Abstract

**Background:**

Bovine viral diarrhoea virus (BVDV), an enveloped, single-stranded, positive-sense RNA virus from the *Flaviviridae* family, is a globally distributed bovine pathogen. BVDV infection in cattle, despite having a wide range of clinical manifestations, is invariably responsible for significant economic losses. To counteract these losses, various schemes to control and eradicate BVDV have been implemented, although safe drugs effectively inhibiting the replication of the virus are still lacking. The purpose of this study was to characterize the antiviral effect of naturally occurring proteins and peptide, such as bovine lactoferrin, chicken egg lysozyme, and nisin from *Lactococcus lactis*, used both individually and in combination, against the cytopathic NADL strain of BVDV in vitro. After determining the cytotoxicity level of each protein or peptide to MDBK cells, its antiviral effects were evaluated using virucidal, cytopathic effect inhibition and viral yield reduction assays. In addition, the influence of the tested compounds on the intracellular viral RNA level was determined.

**Results:**

The highest efficacy among the single treatments was achieved by bovine lactoferrin, which was effective both at the early stages of viral infection and during its entire course, although the effect weakened over time. Nisin and lysozyme were effective at later stages of infection, and the intensity of their effect did not diminish with time. Nisin+lactoferrin and lysozyme+lactoferrin combinations demonstrated stronger antiviral effects than did the single substances. The nisin+lactoferrin mixture present during the whole period of infection produced the strongest anti-BVDV effect in our entire research on both the extracellular viral titre (titre reduction up to 2.875 log ≈ 99.9%) and the intracellular viral RNA level (reduction up to 89%), and this effect intensified over the incubation time.

**Conclusions:**

The tested substances could be applied in bovine viral diarrhoea prevention and therapy, especially when used in combination.

**Electronic supplementary material:**

The online version of this article (10.1186/s12917-019-2067-6) contains supplementary material, which is available to authorized users.

## Background

Bovine viral diarrhoea virus (BVDV), a member of the *Flaviviridae* family, genus *Pestivirus*, is an enveloped, icosahedral, single-stranded, positive-sense RNA virus. Two types of the virus, BVDV1 and BVDV2, are distinguished according to genome differences, while the changes caused in infected cells lead to the distinction between two biotypes: cytopathic (cp) and non-cytopathic (ncp) biotypes [1]. Bovine viral diarrhoea affects cattle populations around the world and causes considerable economic losses due to reproductive disorders, low productivity and higher incidence of other infectious diseases among the affected individuals [2]. The virus can cause acute infections with a mild or asymptomatic course, persistent infections and even fatal mucosal disease, and the virus biotype is crucial for the pathogenesis of an infection [3]. More specifically, only the ncp biotype is responsible for persistent infections, whereas transient infections can be caused by either biotype [4]. Transient infection is associated with the isolation of low virus titres from affected cows and with a slowly growing level of specific antibodies. Infection of a seronegative female with an ncp BVDV strain between 40 and 120 days of gestation leads to the development of immunotolerance in the foetus and to persistent infection. Persistently infected (PI) animals are characterized by the absence of antibody production and the constant presence of high virus titres in the organism [3, 4]. Those individuals act as a reservoir of the virus and maintain it in the population due to constant BVDV shedding in all excretions and secretions [2]. Both biotypes of the virus interfere with the mechanisms of innate and acquired immunity, elevating the affected animal’s predisposition to infections with other pathogens [4]. To reduce economic losses, BVDV control and eradication schemes have been implemented, and these entail identification of affected herds, removal of the virus from a herd by detection and elimination of PI animals, and prevention of re-infection [2]. Vaccination is also practised; however, the presence of neutralizing antibodies does not eliminate virus shedding, nor does it reduce the number of BVD cases [5].

Various natural substances and synthetic compounds have been tested as potential anti-BVDV agents [6–8]. High efficacy in vitro is achieved particularly by nucleoside analogues, but their administration is associated with negative side effects (cytotoxicity, immunosuppression) [6]. Naturally occurring substances (extracts from plants, animal tissues, fungi or bacteria) are characterized by greater biocompatibility and safety. Among the most frequently tested animal proteins are lactoferrin and lysozyme, both of which are factors of innate immunity that are produced in the granules of neutrophils and in the mucosal epithelium and are present in secretions of an organism [9]. Lactoferrin (LF) is a multi-functional glycoprotein with a broad spectrum of antiviral activity against cytomegalovirus (CMV), herpes simplex virus (HSV), human immunodeficiency virus (HIV), hepatitis C virus (HCV), hepatitis B virus, rotavirus, norovirus, poliovirus, respiratory syncytial virus, parainfluenza virus, influenza A virus, hantavirus, human papillomavirus, feline calicivirus, bovine herpesvirus 1 (BoHV-1), murine norovirus, adenovirus, enterovirus 71, echovirus 6, Japanese encephalitis virus, Sindbis virus and Semliki forest virus [9–11]. Lysozyme is an enzyme hydrolysing glycosidic linkages in bacterial peptidoglycan; due to the low toxicity of lysozyme, it is used as a natural preservative to control bacteria in meat products [12]. The antiviral spectrum of lysozyme is considerably more modest than that of lactoferrin and mainly includes HSV1 and HIV-1 [9].

Nisin is a bacteriocin produced by some strains of *Lactococcus lactis* bacterium, a member of a class of thermostable cationic antibacterial peptides comprising atypical amino acid residues forming lanthionine rings called lantibiotics. In 1988, the Food and Drug Administration (FDA) gave nisin a GRAS status (generally regarded as safe). Because nisin has a wide spectrum of antibacterial effects, it is used as a natural food preservative, especially in processed cheese [13]. Similar to lactoferrin and lysozyme, nisin is characterized by a positive charge, which can facilitate electrostatic interactions with the viral capsid [12]. To date, the antiviral activity of numerous bacteriocins produced by lactic acid bacteria has been described, but the antiviral activity of nisin has not been reported [14].

The objective of this study was to characterize the antiviral effects of bovine lactoferrin, chicken egg lysozyme, and nisin from *Lactococcus lactis*, both individually and in combination, on BVDV in vitro. To date, none of the above substances have been tested with respect to their anti-BVDV activity.

## Results

### Compound cytotoxicity

**Fig. 1:**
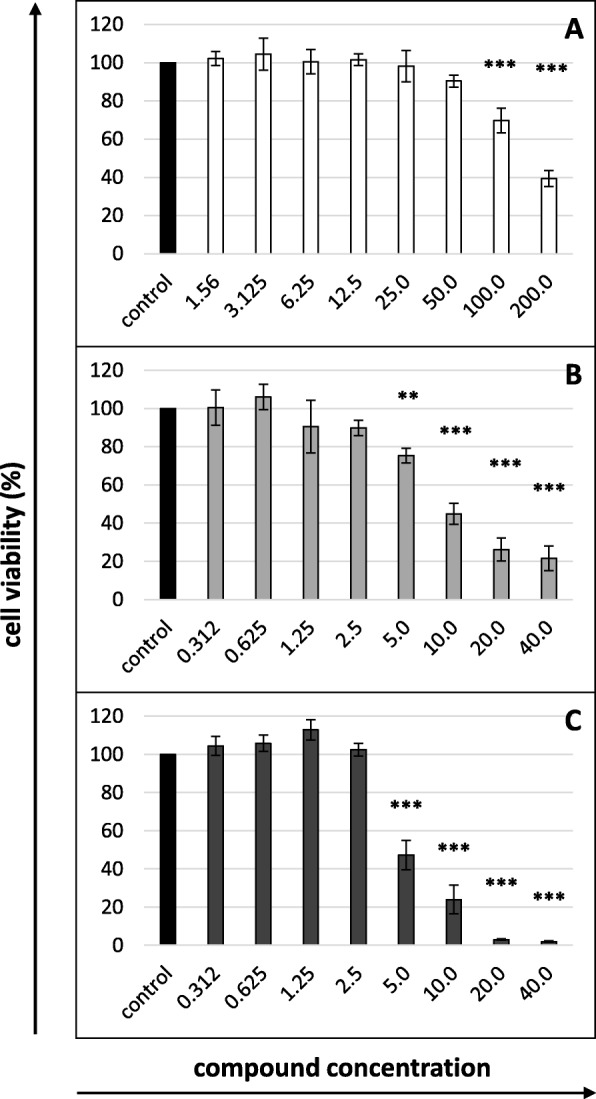
Cytotoxicity of (**a**) nisin, (**b**) lysozyme and (**c**) lactoferrin after 5 days of incubation (MTT assay). Cell viability expressed as the percentage of control (untreated) cell viability. Nisin concentrations in μg/mL, lysozyme and lactoferrin concentrations in mg/mL. All data expressed as means ± SD (standard deviation) for *n* = 3 independent experiments. Asterisks refer to statistically significant differences between control and treatments at: ** *p*<0.01, *** *p*<0.001

The two highest concentrations of nisin (100 and 200 μg/mL) significantly decreased the viability of the MDBK cells after 5 days of incubation (Fig. 1a). Although the viability of cells incubated with nisin at a concentration of 50 μg/ml was 90%, slight changes in the cell morphology were observed under a microscope after 5 days of culture; therefore, the maximum tolerable concentration (MTC) of nisin was assumed to be 25 μg/mL. This concentration and four subsequent concentrations, each twice as low as the previous concentration (12.5, 6.25, 3.125 and 1.56 μg/mL), were selected to test the anti-BVDV activity. The 50% cytotoxic concentration (CC_50_) of nisin equalled 167.275 μg/mL (Table 1).

**Table 1:** CC_50_, EC_50_ and SI values of nisin, lysozyme and lactoferrin

	nisin	lysozyme	lactoferrin
CC_50_	167.275 ± 5.127	9.25 ± 0.836	4.907 ± 0.845
EC_50_	11.916 ± 2.547	0.516 ± 0.312	0.29 ± 0.233
SI	14.0378	17.926	16.92

Nisin concentrations in μg/mL, lysozyme and lactoferrin concentrations in mg/mL. CC_50_ (50% cytotoxic concentration) and EC_50_ values (50% effective concentration) expressed as means ± SD (standard deviation) for *n* = 3 independent experiments. CC_50_ and EC_50_ values were calculated using the ED50 Plus v1.0 online software. Selectivity indices (SI; ratio CC_50_/ EC_50_)

As for lysozyme and lactoferrin, concentrations of the proteins between 5 and 40 mg/mL were toxic to cells (Fig. 1b and c, respectively). Because the viability of cells in the presence of 1.25 mg/mL of lysozyme was exactly 90%, the maximum concentration chosen for the following tests was 1 mg/mL, and four subsequent ones, each twice as low as the previous one, were 0.5, 0.25, 0.125 and 0.06 mg/mL. The CC_50_ of lysozyme was 9.25 mg/mL (Table 1). Although the MTC of lactoferrin was 2.5 mg/mL, concentrations of 1, 0.5, 0.25, 0.125 and 0.06 mg/mL were chosen for further testing. This choice was dictated by the extensive data in the literature, which indicate that lactoferrin typically produces antiviral effects in vitro when present in concentrations below 1 mg/mL. The CC_50_ of lactoferrin was 4.907 mg/mL (Table 1).

Nisin+lactoferrin, lysozyme+lactoferrin and nisin+lysozyme mixtures in the tested concentrations were not toxic to MDBK cells (Additional file 1).

### Cytopathic effect inhibition by the compounds

**Fig. 2:**
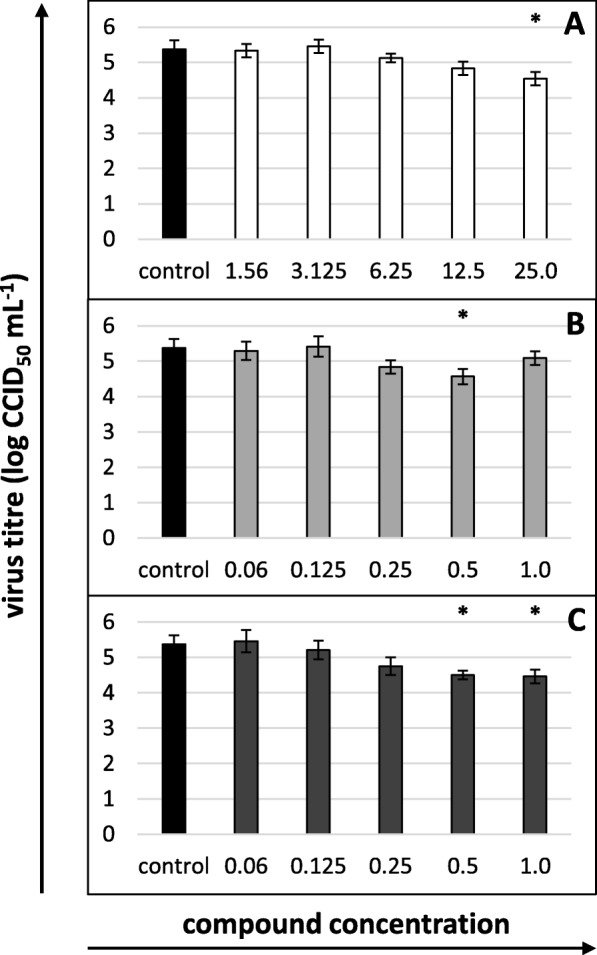
Cytopathic effect inhibition by (**a**) nisin, (**b**) lysozyme and (**c**) lactoferrin after 5 days of incubation. The 50% endpoint virus titres (CCID_50_) were calculated using the Reed and Muench method. Nisin concentrations in μg/mL, lysozyme and lactoferrin concentrations in mg/mL; control: untreated virus. All data expressed as means ± SD (standard deviation) for *n* = 3 independent experiments. Asterisks refer to statistically significant differences between control and treatments at: *p<0.05

Considerably lower final titres were observed for the virus titrated in the presence of nisin MTC (a decrease by 0.833 log, i.e., circa 86%), lysozyme at a concentration of 0.5 mg/mL (by 0.808 log, i.e., circa 85%) or 1 and 0.5 mg/mL of lactoferrin (by 0.917 and 0.875 log, i.e., circa 90 and 88%, respectively) (Fig. 2a, b and c, respectively). The 50% effective concentration (EC_50_) of nisin was 11.916 μg/mL, that of lysozyme was 0.516 mg/mL, and that of lactoferrin was 0.29 mg/mL. The selectivity index (SI) was similar for all the substances and equalled 14.038, 17.926 and 16.92, respectively (Table 1).

**Fig. 3:**
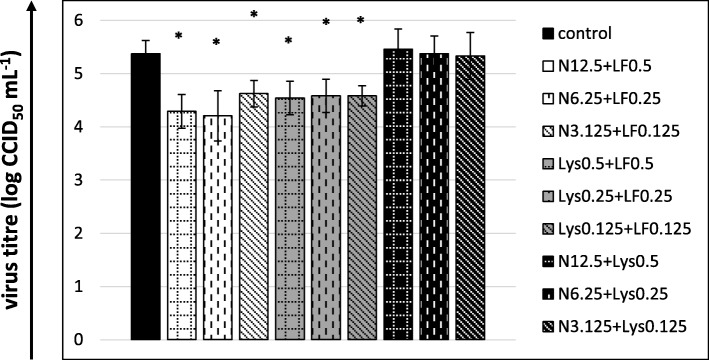
Cytopathic effect inhibition by nisin+lactoferrin, lysozyme+lactoferrin and nisin+lysozyme mixtures after 5 days of incubation. The 50% endpoint virus titres (CCID_50_) were calculated using the Reed and Muench method. Nisin concentrations in μg/mL, lysozyme and lactoferrin concentrations in mg/mL; control: untreated virus. All data expressed as means ± SD (standard deviation) for *n* = 3 independent experiments. Asterisks refer to statistically significant differences between control and treatments at: *p<0.05

Two treatment mixtures, nisin+lactoferrin and lysozyme+lactoferrin, used in any of the tested concentrations, were able to considerably decrease the final titre of the virus. The effect of the nisin+lactoferrin combination manifested itself more strongly (a decrease by 1.083, 1.167 and 0.75 log, i.e. ca. 90.5, 91 and 82%) than that of the lysozyme+lactoferrin mixture (a decrease by 0.833 at the highest concentration and 0.792 log at the two other concentrations, i.e. ca. 85 and 83%) (Fig. 3). The mixture of nisin+lysozyme did not affect the final titre of the virus (Fig. 3).

### Virucidal activity of the tested treatments

None of the tested single substances, nor any of their mixtures, had a significant influence on the titre of the virus after direct contact with virus particles, regardless of the time or temperature of the contact (Additional file 2).

### Viral yield reduction by the treatments – determination of the viral infection stage

**Fig. 4:**
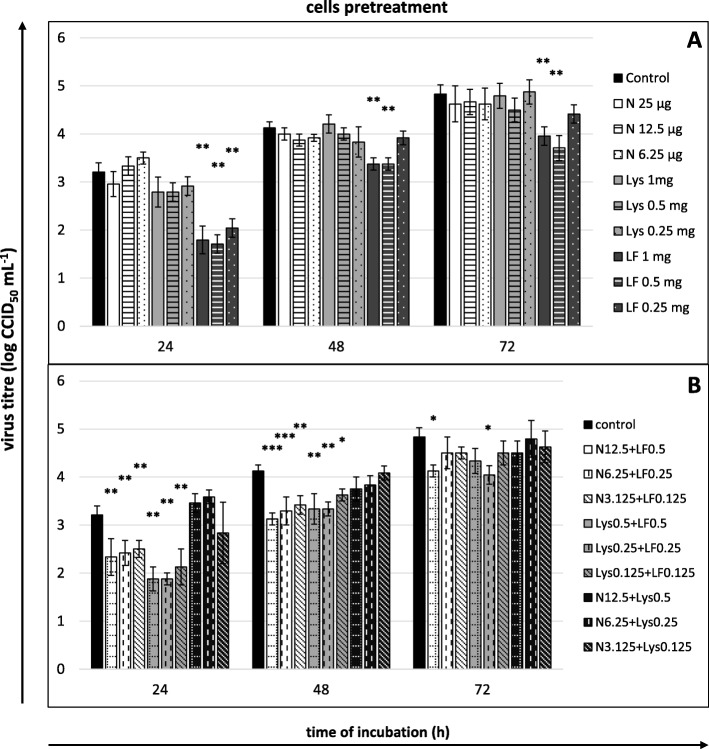
Viral yield reduction – cells pretreatment. Cells pretreated for 2 h before infection with (**a**) single compounds or (**b**) their mixtures. Culture supernatants collected for virus titration (CCID_50_) after 24, 48 and 72 h of incubation. Nisin (N) concentrations in μg/mL, lysozyme (Lys) and lactoferrin (LF) concentrations in mg/mL; control: untreated cells. All data expressed as means ± SD (standard deviation) for *n* = 3 independent experiments. Asterisks refer to statistically significant differences between control and compound-treated virus at: *p<0.05, ** *p*<0.01, *** *p*<0.001

**Fig. 5:**
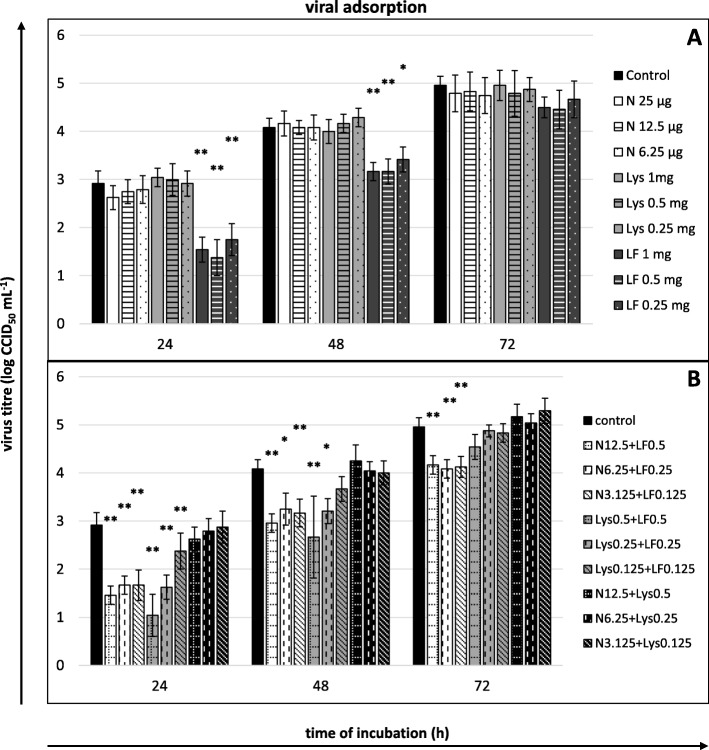
Viral yield reduction - viral adsorption. **a** compounds or **b** their mixtures present only during viral adsorption (1 h). Culture supernatants collected for virus titration (CCID_50_) after 24, 48 and 72 h of incubation. Nisin (N) concentrations in μg/mL, lysozyme (Lys) and lactoferrin (LF) concentrations in mg/mL; control: untreated cells. All data expressed as means ± SD (standard deviation) for *n* = 3 independent experiments. Asterisks refer to statistically significant differences between control and compound-treated virus at: *p<0.05, ** *p*<0.01

**Fig. 6:**
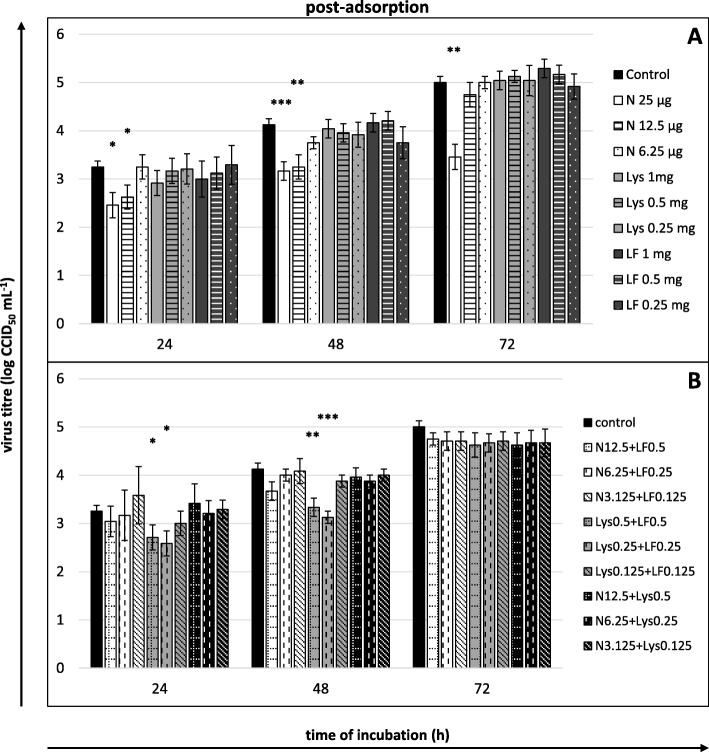
Viral yield reduction - post-adsorption stage. **a** compounds or **b** their mixtures present only after viral adsorption. Culture supernatants collected for virus titration (CCID_50_) after 24, 48 and 72 h of incubation. Nisin (N) concentrations in μg/mL, lysozyme (Lys) and lactoferrin (LF) concentrations in mg/mL; control: untreated cells. All data expressed as means ± SD (standard deviation) for *n* = 3 independent experiments. Asterisks refer to statistically significant differences between control and compound-treated virus at: *p<0.05, ** *p*<0.01, *** *p*<0.001

**Fig. 7:**
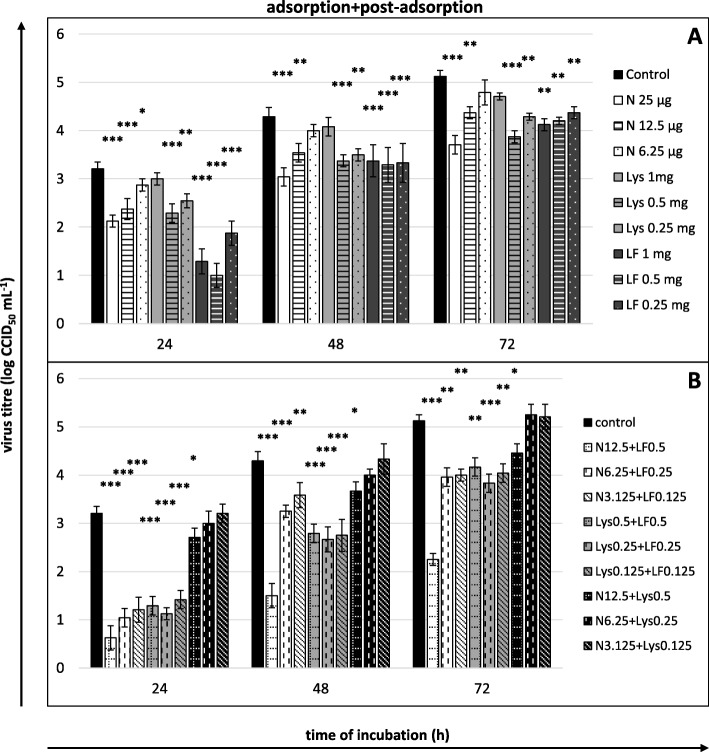
Viral yield reduction - the entire course of infection. **a** compounds or **b** their mixtures present both during and after adsorption. Culture supernatants collected for virus titration (CCID_50_) after 24, 48 and 72 h of incubation. Nisin (N) concentrations in μg/mL, lysozyme (Lys) and lactoferrin (LF) concentrations in mg/mL; control: untreated cells. All data expressed as means ± SD (standard deviation) for *n* = 3 independent experiments. Asterisks refer to statistically significant differences between control and compound-treated virus at: *p<0.05, ** *p*<0.01, *** *p*<0.001

Lactoferrin was the only substance that produced effects at the stages of cell preincubation and viral adsorption, and the intensity of this influence weakened as the incubation time lengthened (Fig. 4a and 5a). No inhibitory effect of lactoferrin was observed at the post-adsorption stage (Fig. 6a), but when the protein was present in the culture medium throughout that the whole duration of infection (adsorption+post-adsorption), it had the strongest effect. In the latter conditions, a decrease in the titre of the virus was noted during the entire experiment and at all concentrations of lactoferrin (Fig. 7a). The most profound effect, irrespective of which experimental variant was analysed, occurred at the early stage of infection (24 h) at the lactoferrin concentration of 0.5 mg/mL (reduction of the titre at the preincubation stage by 1.5 log, at the adsorption stage by 1.542 log, and at the adsorption+post-adsorption stage by 2.208 log, that is, by approximately 96.8, 97.2 and 99.4%, respectively).

Nisin reduced the viral yield at the post-adsorption stage and when present in the culture medium throughout the entire course of infection (adsorption+post-adsorption), and the intensity of the effect was proportional to the concentration of nisin. Lower concentrations were effective only during the first 24–48 h after inoculation. It was only the highest concentration of nisin (25 μg/mL) that remained effective throughout the entire experiment, and the intensity of its influence increased with the time of incubation (maximum reduction of the titre after 72 h, reaching 1.542 log at the post-adsorption stage, 1.417 log, at the adsorption + post-adsorption stage, i.e., by approximately 97 and 96%, respectively) (Fig. 6a and 7a).

Lysozyme reduced the viral yield only when present in the culture medium throughout the entire duration of infection (adsorption+post-adsorption). A significant reduction of extracellular virus titre was observed at lysozyme concentrations of 0.5 and 0.25 mg/mL during the entire experiment, and as in the case of nisin, the intensity of the effect produced by lysozyme at 0.5 mg concentration was the highest after 72 h of incubation (reduction of the titre by 1.25 log, i.e., by approximately 94%; Fig. 7a).

The mixtures of nisin+lactoferrin and lysozyme+lactoferrin in concentrations of half the single substance doses were distinguished by high efficacy. Nisin with lactoferrin considerably reduced the viral yield at the stages of cell preincubation, viral adsorption and adsorption+post-adsorption, whereas lysozyme with lactoferrin produced such effects in all experimental variants (Fig. 4b, 5b, 6b and 7b). Similar to individual treatments, these mixtures led to effects that were proportional to the applied concentrations and that decreased with the incubation time. Only the intensity of the effect induced by the highest nisin+lactoferrin mixture concentration at the adsorption+post-adsorption stage intensified during the incubation time. This mixture concentration also caused the strongest anti-BVDV effect in our entire research (titre reduction by 2.583 log after 24 h, 2.792 log after 48 h and by 2.875 log after 72 h, i.e., by approximately 99.7, 99.8 and 99.9%, respectively).

The mixture of nisin and lysozyme was characterized by the weakest efficacy. This mixture reduced the viral titre only at the adsorption+post-adsorption stage and only when applied at the highest tested concentration (Fig. 7b).

### Treatment effect on viral RNA synthesis in BVDV-infected cells

**Fig. 8:**
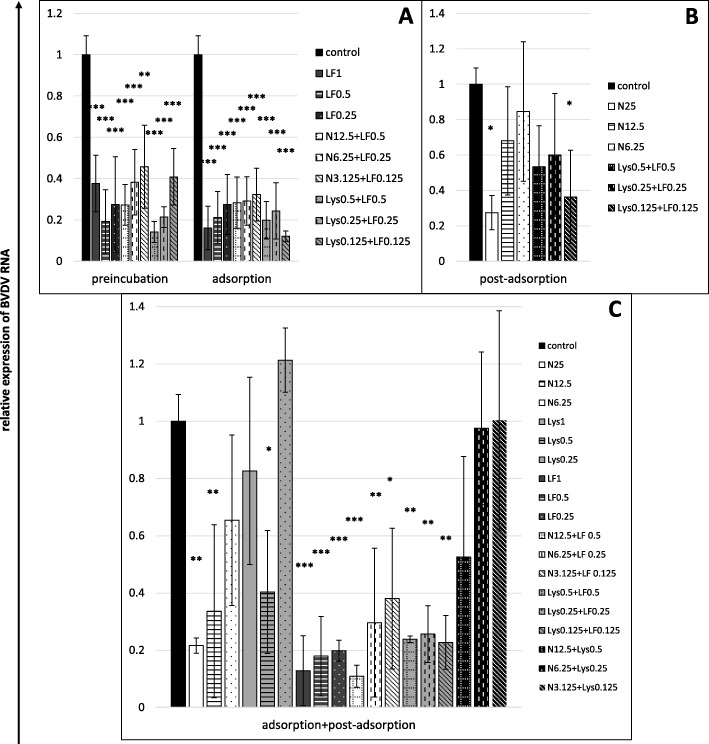
Treatment effect on viral RNA synthesis in BVDV-infected cells after 24 h of infection (RT-qPCR). **a** single compounds or their mixtures present for 2 h before infection (preincubation) or during viral adsorption, **b** single compounds or their mixtures present only after viral adsorption, **c** single compounds or their mixtures present both during and after adsorption. Amounts of intracellular BVDV RNA from control (compound-untreated) cells set as 1, results obtained from treated cells expressed as the relative amount of the control virus RNA. Nisin (N) concentrations in μg/mL, lysozyme (Lys) and lactoferrin (LF) concentrations in mg/mL. All data expressed as means ± SD (standard deviation) for *n* = 3 independent experiments. Asterisks refer to statistically significant differences between control and compound-treated virus at: *p<0.05, ** *p*<0.01, *** *p*<0.001

Although the tested substances and their mixtures had a weaker effect on the amounts of the intracellular viral RNA than on extracellular virus titres, the results of both tests correspond to each other. A higher reduction of the RNA amounts was observed at the stages of cell preincubation and viral adsorption than at the stage of post-adsorption, and the intensity of the effect tended to be proportional to the applied concentrations of the tested compounds (Fig. 8a and b). Similar to the extracellular virus titres, the strongest effect was observed at the highest concentration of the nisin+lactoferrin mixture at the adsorption+post-adsorption stage (a decrease in the intracellular RNA amount by 89%; Fig. 8c). The nisin+lysozyme mixture did not decrease the synthesis of the BVDV RNA, irrespective of the applied concentration (Fig. 8c).

## Discussion

The study described in this paper is one of the few to investigate the antiviral effect of nisin to date. This bacteriocin decreased both the extracellular virus titre and the amount of intracellular viral RNA. The best effect was observed when nisin was present throughout the entire duration of viral infection (adsorption+post-adsorption), although the application of the peptide only at the post-adsorption stage also produced a desirable effect, and its intensity did not weaken with the incubation time.

The potential antiviral activity of nisin has been tested mainly in the context of its virucidal activity in food. In the experiment by Ly-Chatain et al. [12], only very high concentrations of nisin (100,000 IU, i.e., 25 mg) after 10 min of contact decreased the titres of bacteriophages c2 and MS2 by 1 log. In the research performed by Lange-Starke et al. [15], nisin did not demonstrate any effect against murine norovirus S99, influenza A virus A/WSN/33, Newcastle disease virus Montana and feline herpesvirus KS 285, even after a three-day contact at a temperature of 24 °C. These results agree with the lack of antiviral activity of nisin shown in our experiment (concentrations up to 25 μg/mL, contact time up to 60 min, range of temperatures 4–37 °C).

The only experiment in which the anti-cytomegalovirus effect of nisin was tested in the cytopathic effect inhibition assay verified the antiviral activity of nisin, showing an IC_50_ (inhibitory concentration) of 255 μg/mL. The CC_50_ concentration of nisin against human foetal lung fibroblasts was > 2 mg/mL. Unfortunately, the researchers did not state explicitly at which stage of a viral infection nisin was effective [16]. In our study, the EC_50_ of nisin was much lower (approximately 12 μg/mL), similar to its CC_50_ (167 μg/mL). A possible cause of these discrepancies is a different level of susceptibility of the model cells and the virus used in both studies.

Lysozyme was characterized by the weakest anti-BVDV effect among all the tested compounds. Similar to nisin, lysozyme did not enter into direct interactions with the virus or with the cell, nor did it inhibit viral adsorption. To reduce the virus titre and viral RNA amount, the enzyme had to be present throughout the whole course of viral infection (adsorption+post-adsorption), and the intensity of its impact increased with the incubation time. Most of the available literature focuses on the antiviral activity of lysozyme against herpes simplex virus and human immunodeficiency virus [9], although there are also reports negating the anti-HSV-1 effect of this enzyme [17, 18]. The anti-HIV-1 activity of lysozyme is ascribed to its direct interaction with the virus (the blockade of viral fusion protein gp41) or with the cell (the blockade of CD4 receptor on lymphocytes) [19]. It was only in the experiment by Zhang et al. [20] that lysozyme produced by the marine strain of bacteria from the genus *Bacillus* inhibited the replication of pseudorabies virus in porcine kidney cells PK-15, not via direct interactions with the virus or the cell but during/after the infection of a cell, which is in keeping with our results. The CC_50_ and EC_50_ concentrations of the bacterial lysozyme were considerably lower (100 μg/mL and 0.46 μg/mL, respectively) than the analogous concentrations achieved in our experiment (9.25 mg/mL and 0.516 mg/mL).

Bovine lactoferrin was characterized by the broadest spectrum of action among all tested compounds. Similar to the other proteins, LF did not demonstrate virucidal activity, but it was the only substance that had a protective effect on the cell and inhibited the adsorption of the virus. Interestingly, LF also produced a strong effect when it was present during the entire course of infection, despite being inactive at the post-adsorption step alone. Unlike nisin and lysozyme, the anti-BVDV effect of lactoferrin weakened with time. The results obtained in our experiment agree with the wealth of data in the literature concerning the antiviral activity of LF, which suggest that the protein usually prevents viral infection at its early stage by binding directly to the viral particles or to the cell [10, 11]. Depending on an experimental model, different ranges of effective concentrations of lactoferrin have been identified in in vitro studies, although EC_50_ typically was no more than 1 mg/mL [10], as in our experiment. Fewer papers also implicate lactoferrin can inhibit the intracellular stages of viral replication. For example, Välimaa et al. [18] confirmed the antiviral activity of LF against HSV-1 at different replication cycle stages (preincubation with virus or cells, adsorption and post-adsorption), with EC_50_ values before adsorption being lower (100–250 μg/mL) than those observed after the adsorption of the virus (250–500 μg/mL).

The only bovine virus against which the antiviral activity of lactoferrin has been tested is bovine herpesvirus 1 which, similar to BVDV, is widespread in cattle populations [21]. In the cited study, however, considerably higher concentrations of lactoferrin (1.25–10 mg/mL) were tested than in our experiment. The concentration of 10 mg/mL decreased the amount of PFU BoHV-1 by 99%, while lower concentrations caused a 90–99% reduction. According to the quoted authors, this range of concentrations was not toxic to MDBK cells after 120 h of incubation, whereas in our study, the CC_50_ of lactoferrin was 4.9 mg/mL, and the concentration of 10 mg/mL lowered the viability of MDBK cells by over 75%.

The activity of bovine lactoferrin against hepatitis C virus has also been described. BVDV is sometimes used in research as a surrogate model of HCV replication in vitro. Both of these viruses belong to the same family and share many structural, functional and genomic features. However, the propagation of HCV in vitro is a highly demanding undertaking, while BVDV culture is rapid and easy to perform [22]. The mechanism of action of lactoferrin against BVDV demonstrated in our study differed from the mechanism of its action against HCV described by Ikeda et al. [23]. LF inhibited the HCV entrance into human hepatocytes and T lymphocytes owing to the direct interaction with the virus, while preincubation of cells or addition of the protein after inoculation produced no effect. Inactivation of HCV was an effect of LF interacting with the viral envelope proteins (E1 and E2). It is possible that the similarity between HCV and BVDV is insufficient for a similar mechanism of lactoferrin’s antiviral activity to arise, since both viruses belong to two different genera of *Flaviviridae* family, *Pestivirus* (BVDV) and *Hepacivirus* (HCV).

The nisin+lactoferrin and lysozyme+lactoferrin mixtures were distinguished by being more effective against BVDV than single proteins, despite being tested in lower concentrations. This result was unsurprising because it is known that co-administration of substances with different mechanisms of action can intensify their activity and allow one to decrease a therapeutic dose which, in turn, helps to minimize side effects [9, 11]. Nisin and lysozyme are safe natural food preservatives, while lactoferrin is a safe commercial nutraceutical. All of these compounds could be an alternative to synthetic antimicrobials, either as substitutes or as supplementary treatments in the standard therapy, and LF as a modulator of the immune system could also have a positive influence on the course of infection by enhancing the immunological response of the host [9–11, 13], and this effect may be particularly important in the case of viruses causing immunosuppression, such as BVDV. To the best of our knowledge, these proteins and peptide have not been tested in combination to determine their antiviral effects.

## Conclusions

All the tested substances demonstrated anti-BVDV effects in vitro, although the effects varied in intensity and at different steps of the infection of cells. The highest efficacy among the single treatments was shown by bovine lactoferrin, which was effective at both the early steps and throughout the entire course of infection. However, its effects waned with time. Nisin and lysozyme were effective at later steps of infection than lactoferrin, and the intensity of their influence did not decrease with time. The nisin+lactoferrin and lysozyme+lactoferrin mixtures resulted in a more intensive antiviral effect at lower concentrations of the tested substances. The nisin+lactoferrin mixture present throughout the whole course of infection produced the strongest anti-BVDV effect in our entire research on both the extracellular viral titre and the intracellular viral RNA level, and its impact on the viral yield grew stronger with the time of incubation. Owing to the low toxicity and decent selectivity index of the tested substances, they could be used in prophylaxis (protecting the cell) or in BVDV therapy (inhibition of viral replication), especially in combination.

## Methods

### Cells and virus

Madin-Darby bovine kidney (MDBK, ATCC CCL-22) cells were grown in Dulbecco’s modified Eagle’s medium (DMEM) supplemented with 10% horse serum, 1% non-essential amino acid solution and 1% antibiotic-antimycotic solution (all reagents purchased from Sigma-Aldrich, Germany) at 37 °C in a humidified atmosphere with 5% CO_2_.

The cytopathic strain of bovine viral diarrhoea virus 1 (NADL strain, ATCC VR-534) was propagated and titrated in MDBK cells and stored at − 80 °C until use. The cells were seeded in 96-well plates at a density of 1 × 10^5^ cells/mL and grown for 24 h before virus titration to achieve approximately 60–70% confluence. Then, 100 μl of 10-fold serial dilutions of virus (10^− 1^–10^− 7^) were added to each well (eight wells per dilution). Five days after infection, the cytopathic effect was recorded using an inverted phase contrast microscope. The 50% endpoint virus titres (CCID_50_, 50% cell culture infective dose) were calculated using the Reed and Muench method [24].

### Compounds

Nisin from *Lactococcus lactis*, lysozyme from chicken egg white, and lactoferrin from bovine milk, all of which were purchased from Sigma-Aldrich, were tested for their anti-BVDV activity. A stock solution of nisin was prepared in ultrapure water (Milli-Q A10, Millipore, France) at a concentration of 2 mg of pure nisin per mL, and working solutions were prepared in a cell maintenance medium at final concentrations of 0 (control cells), 1.56, 3.125, 6.25, 12.5, 25, 50, 100 and 200 μg/mL. Lysozyme and lactoferrin were dissolved straight in DMEM to reach final protein concentrations of 0 (control cells), 0.312, 0.625, 1.25, 2.5, 5, 10, 20 and 40 mg/mL.

### Cytotoxicity assay

The cytotoxicity of the tested substances was determined using the MTT reduction colorimetric assay after 5 days of cell culture according to the previously described protocol [25]. The viability of treated cells was expressed as the percentage of control (untreated) cell viability. All experiments were repeated three times. CC_50_ values (50% cytotoxic concentration, decreasing cell viability by 50%) were calculated using the ED50 Plus v1.0 online software. The concentrations of tested compounds that resulted in a reduction in cell viability by less than 10% were regarded as the maximum tolerable concentrations (MTC) and selected for further testing.

### Cytopathic effect inhibition assay

To confirm the potential anti-BVDV activity of the tested compounds, their effects on the final virus titre were evaluated. The virus was titrated in the presence of different nontoxic concentrations of the tested compounds, as described above (paragraph Cells and virus). The control virus was titrated in a compound-free medium. All experiments were repeated three times. EC_50_ values (50% effective concentration, decreasing the final virus titre by 50%) were calculated using the ED50 Plus v1.0 online software. The selectivity index for each compound was calculated by dividing their CC_50_ values by EC_50_ values.

In the next step, the effects of compound combinations (nisin+lactoferrin, lysozyme+lactoferrin and nisin+lysozyme) on the final virus titre were also evaluated. Compounds in mixtures were tested in three concentrations: half, one fourth and one eighth the MTC. Before testing, each combination was checked for cytotoxicity. All experiments were repeated three times.

### Virucidal assay

To evaluate potential direct virus inactivation by the compounds (virucidal activity), the stock virus was placed into contact with MTC of the compound in DMEM (the stock virus final dilution 1:10) under different experimental conditions (contact times 10 or 60 min; contact temperatures 4, 20 or 37 °C). The highest concentrations of the compound mixtures described above (paragraph Cytopathic effect inhibition assay) were also tested. The control virus was diluted with compound-free medium and incubated under the same conditions. Each mixture was then titrated in MDBK cells, and CCID_50_ titres of treated virus were compared with control virus titres (tested under the same set of conditions). Each experiment was repeated three times.

### Yield reduction assay

To determine the mode of antiviral action of the tested compounds, MDBK cells were seeded in 24-well plates at a density of 1 × 10^5^ cells/well and grown for 24 h before infection. Then, the growth medium was replaced with a maintenance medium containing BVDV at an MOI (multiplicity of infection) of 0.1 and incubated at 37 °C for 1 h. Afterwards, the inoculum was removed, cells were washed twice with PBS, and fresh maintenance medium was added to wells. Three different treatments with the tested compounds (in the maximum tolerable concentrations and half and one fourth the MTC) were carried out:

- cells pretreated for 2 h before infection.
- compounds present only during viral adsorption (1 h).
- compounds present only after adsorption.
- compounds present both during and after adsorption.

After 24, 48 and 72 h of incubation, culture supernatants were collected, and the extracellular virus was titrated (CCID_50_) in MDBK cells, as described previously (paragraph Cells and virus). The compound mixtures were also tested.

All experiments were repeated three times.

### RNA isolation and RT-qPCR

Only those treatments/experimental designs (paragraph Yield reduction assay) which decreased the extracellular virus titre were further tested for their effects on the intracellular BVDV RNA synthesis after 24 h. For this purpose, after 24 h incubation, the supernatants were removed, cells were washed twice with PBS, and 0.8 ml of Fenozol reagent (A & A Biotechnology, Poland) was added to each well and mixed by pipetting until complete cell lysis occurred.

RNA was extracted using a Total RNA Mini kit (A&A Biotechnology, Poland) according to the manufacturer’s protocols. Eluted RNA concentrations were measured using a BioSpectrometer® (Eppendorf, Hamburg, Germany) and stored at − 80 °C for further analysis.

Real-time PCR analysis was performed using a Bovine Viral Diarrhoea Virus Advanced Kit (PrimerDesign, Chandler’s Ford, United Kingdom) according to the manufacturer’s protocol. Amplification reactions were carried out with a LightCycler® 96 Real-Time PCR thermocycler (Roche, Meylan, France).

Intracellular viral RNA detected from BVDV-infected untreated (control) cells was assumed to equal 1, and the results obtained from treated cells were expressed as the relative amount of the control virus RNA. Each experiment was repeated three times.

### Statistical analysis

All the results were expressed as the mean values ± standard deviation (SD) of three independent experiments. Data were submitted to one-way analysis of variance (ANOVA). Bonferroni’s post-test was used to determine differences between control and treated cells or the virus. Statistical evaluation of the results was performed using GraphPad Prism software.

## Additional files

Additional file 1: Cytotoxicity of nisin+lactoferrin, lysozyme+lactoferrin and nisin+lysozyme mixtures after 5 days of incubation (MTT assay). Cell viability expressed as the percentage of control (untreated) cell viability. Nisin (N) concentrations in μg/mL, lysozyme (Lys) and lactoferrin (LF) concentrations in mg/mL. All data expressed as means ± SD (standard deviation) for *n* = 3 independent experiments. (DOCX 120 kb)

Additional file 2: Virucidal activity of (**A**) single compounds or (**B**) their mixtures. The stock virus was placed into contact with MTC of the single compounds or the highest concentrations of the compound mixtures under different experimental conditions (contact times 10 or 60 min; contact temperatures 4, 20 or 37 °C). Each mixture was then titrated, and CCID_50_ titres of treated virus were compared with control virus titres (tested under the same set of conditions). Nisin (N) concentrations in μg/mL, lysozyme (Lys) and lactoferrin (LF) concentrations in mg/mL. All data expressed as means ± SD (standard deviation) for *n* = 3 independent experiments. (DOCX 30 kb)

## Data Availability

All data generated or analysed during this study are available from the corresponding author on reasonable request.

## Abbreviations

BVDV: Bovine viral diarrhoea virus
CC_50_: 50% cytotoxic concentration
CCID_50_: 50% cell culture infective dose
cp: cytopathic
DMEM: Dulbecco’s modified Eagle’s medium
EC_50_: 50% effective concentration
LF: Lactoferrin
MDBK: Madin-Darby bovine kidney
MOI: Multiplicity of infection
MTC: Maximum tolerable concentration
ncp: non-cytopathic
RT-qPCR: Real-time quantitative polymerase chain reaction
SI: Selectivity index
