# Supplementary material for: Antiviral effects of nisin, lysozyme, lactoferrin and their mixtures against bovine viral diarrhoea virus
Source: BMC Vet Res. 2019 Sep 5;15:318. doi: 10.1186/s12917-019-2067-6 (PMC6727482; doi:10.1186/s12917-019-2067-6)
Supplement: Supplementary file 1 [file 12917_2019_2067_MOESM1_ESM.docx]

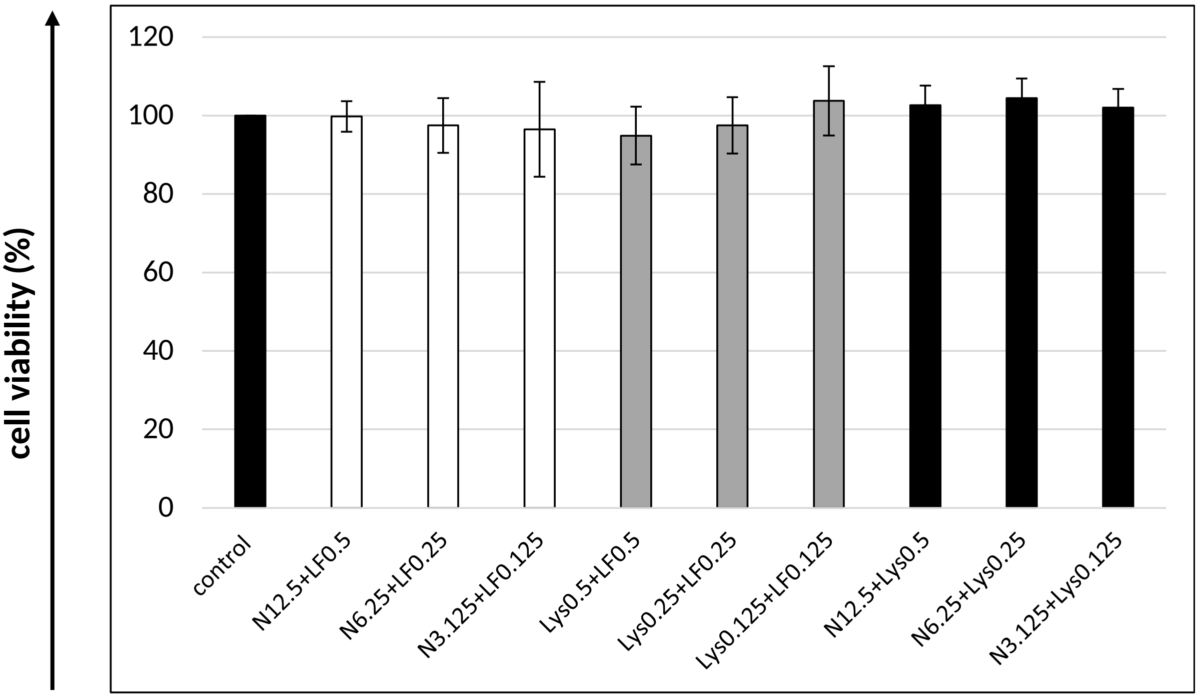
cell viability (%)
120
100
80
60
40
20
0
control
N12.5+LF0.5
N6.25+LF0.25
N3.125+LF0.125
Lys0.5+LF0.5
Lys0.25+LF0.25
Lys0.125+LF0.125
N12.5+Lys0.5
N6.25+Lys0.25
N3.125+Lys0.125
