# Supplementary material for: Antiviral effects of nisin, lysozyme, lactoferrin and their mixtures against bovine viral diarrhoea virus
Source: BMC Vet Res. 2019 Sep 5;15:318. doi: 10.1186/s12917-019-2067-6 (PMC6727482; doi:10.1186/s12917-019-2067-6)
Supplement: Supplementary file 2 [file 12917_2019_2067_MOESM2_ESM.docx]

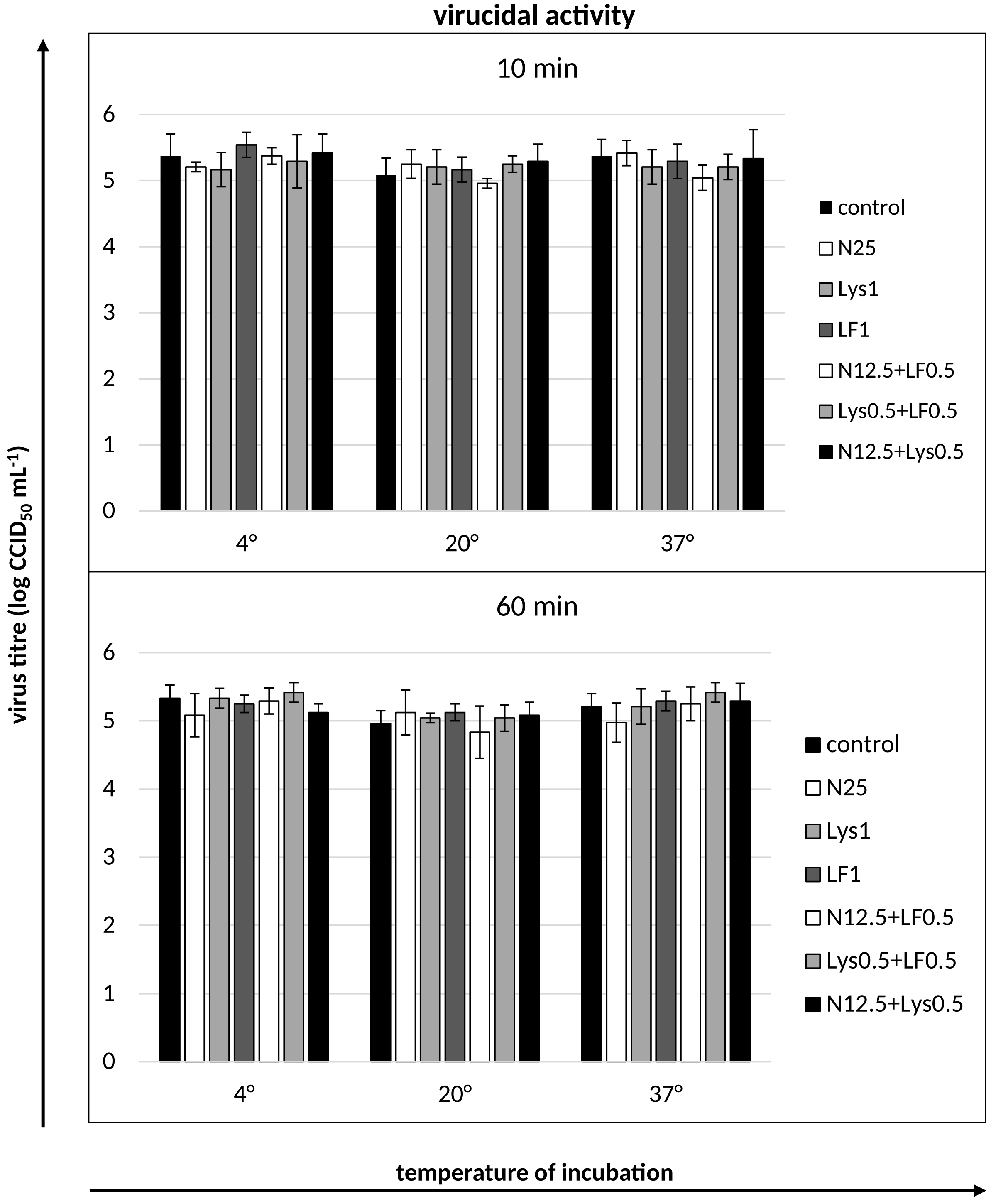

virucidal activity
10 min
6
5
4
3
2
1
0
4°
20°
37°
control
N25
Lys1
LF1
N12.5+LF0.5
Lys0.5+LF0.5
N12.5+Lys0.5
60 min
6
5
4
3
2
1
0
4°
20°
37°
control
N25
Lys1
LF1
N12.5+LF0.5
Lys0.5+LF0.5
N12.5+Lys0.5
virus titre (log CCID50 mL-1)
temperature of incubation
